# Actin remodeling driven by circLIMA1: sperm cell as an intriguing cellular model

**DOI:** 10.7150/ijbs.76261

**Published:** 2022-08-08

**Authors:** Francesco Manfrevola, Nicoletta Potenza, Teresa Chioccarelli, Armando Di Palo, Chiara Siniscalchi, Veronica Porreca, Arcangelo Scialla, Vincenza Grazia Mele, Giuseppe Petito, Aniello Russo, Antonia Lanni, Rosalba Senese, Giulia Ricci, Riccardo Pierantoni, Rosanna Chianese, Gilda Cobellis

**Affiliations:** 1 Department of Experimental Medicine, University of Campania “Luigi Vanvitelli”, 80138 Naples, Italy.; 2 Department of Environmental, Biological, Pharmaceutical Sciences and Technologies, University of Campania “Luigi Vanvitelli”, 81100 Caserta, Italy.

**Keywords:** circRNAs, CB1, actin, spermatozoa, QKI, Gelsolin

## Abstract

CircRNA cargo in spermatozoa (SPZ) participates in setting cell quality, in terms of morphology and motility. Cannabinoid receptor CB1 activity is correlated with a proper spermatogenesis and epididymal sperm maturation. Despite CB1 promotes endogenous skill to circularize mRNAs in SPZ, few notions are reported regarding the functional link between endocannabinoids and spermatic circRNA cargo. In CB1 knock-out male mice, we performed a complete dataset of spermatic circRNA content by microarray strategy.

Differentially expressed (DE)-circRNAs, as a function of genotype, were identified. Within DE-circRNAs, we focused the attention on circLIMA1, as putative actin-cytoskeleton architecture regulator. The validation of circLIMA1 dependent-competitive endogenous RNA (ceRNA) network (ceRNET) in *in vitro* cell line confirmed its activity in the regulation of the cytoskeletal actin. Interestingly, a dynamic actin regulation in SPZ nuclei was found during their epididymal maturation.

In this scenario, we showed for the first time an intriguing sperm nuclear actin remodeling, regulated *via* a ceRNET-independent pathway, consisting in the nuclear shuttling of circLIMA1-QKI interactome and downstream in Gelsolin regulation. In particular, the increased levels of circLIMA1 in CB1 knock-out SPZ, associated with an inefficient depolymerization of nuclear actin, specifically illustrate how endocannabinoids, by regulating circRNA cargo, may contribute to sperm morpho-cellular maturation.

## Introduction

CircRNAs are covalently closed RNA molecules produced by a backsplicing 1. Unlike canonical splicing, during circRNA biogenesis a downstream splice donor site is covalently linked to an upstream splice acceptor site to produce the circular molecules 2. Several RNA binding proteins (RBPs) actively participate for a successful circRNA formation; indeed, recent findings have shown that RNA polymerase II (RNApol2), Quaking (QKI) and Fused protein in Sarcoma (FUS) positively enhance and regulate circRNA biogenesis 3-6.

The primary biological effect exerted by circRNAs consists in the microRNA (miRNA) inhibition by a tethering activity, in order to protect the mRNA targets of miRNAs from degradation 1, 7. In this way, circRNAs take part to a competitive endogenous RNA (ceRNA) network (ceRNET) involved in the regulation of several cellular functions 7, 8.

Despite the growing interest in the study of this new class of RNA molecules, few notions have been reported regarding the involvement of circRNAs in male reproduction. CircRNAs have been identified in human and mouse testis, as well as in spermatogenic cells, with a distribution pathway tightly correlated to germ cell progression and sexual development 9-12. Only recently, a deep characterization of total circRNA cargo in human spermatozoa (SPZ) has been performed, identifying a differential circRNA expression profile related to sperm good and poor quality parameters, in terms of morphology and motility 12. In addition, a circRNA profile has been drawn in asthenozoospermic patients in order to highlight circRNAs as new potential biomarkers of sperm quality, beyond classical morphological parameters 13.

Endocannabinoids are cell-to-cell signaling mediators. The main endocannabinoids characterized in testis and epididymis are anandamide (AEA) and 2-arachidonoylglycerol (2-AG) that exert their biological effects binding the cannabinoid receptor type-1 and type-2 (CB1 and CB2) 14-16. In particular, CB1 receptor activity is required for steroidogenesis and Leydig cell differentiation, as well as spermiogenesis and epididymal sperm maturation 17, 18. The recent evidence regarding the involvement of CB1 receptor in spermatogenesis and male reproduction derived from the study of CB1 knock-out (CB1^-/-^) mouse model that is characterized by: *i*) down-regulation of the hypothalamus-pituitary-gonad (HPG) axis, *ii*) low plasma levels of testosterone and 17β-estradiol, *iii*) production of SPZ with uncondensed chromatin due to abnormal histone retention, highly damaged DNA and elongated nuclear size 18-21.

Endocannabinoid contribution to sperm circRNA content is under-estimated. A clear link between these two classes of biological molecules has been recently shown by our group; indeed a specific circRNA (circNAPEPLDiso1), derived from the linear NAPEPLD transcript, has been found in both human and mouse SPZ and increased in murine fertilized oocytes 11. Interestingly, NAPEPLD is the main enzyme involved in the biosynthesis of AEA 22.

The presence of the specific circNAPEPLDiso1 isoform in SPZ, hypothesized to be paternally transferred to the oocyte, strongly strengthens the idea that endocannabinoids may support sperm circRNA content 11. Accordingly, CB1 involvement in circRNA biogenesis has also been demonstrated in both human and mouse SPZ. Indeed, using CB1^-/-^ male mouse model, an endogenous skill to circularize mRNAs has been found in isolated SPZ and this feature is improved under CB1 stimulation 6.

Based on these intriguing observations, in the current work, we decided to extend our knowledge about the putative functional link between endocannabinoids and spermatic circRNA content. A circRNA microarray analysis was carried out on total epididymal SPZ collected from wild-type (WT) and CB1^-/-^ mice, in order to identify differentially expressed (DE)-circRNAs as a function of genotype. Gene ontology (GO) analysis by using bioinformatic tool showed that circRNA host genes participate to a large number of biological processes concerning cytoskeletal function and remodeling as: *i*) actin filament-based movement, *ii*) actin filament bundle assembly, *iii*) regulation of actin filament organization. Interestingly, within the circRNAs up-regulated in CB1^-/-^ SPZ, we focused our attention on circLIMA1, as its linear counterpart encodes for an actin-binding protein involved in the cytoskeleton regulation and stability by the inhibition of actin filament depolymerization 23, 24.

By a luciferase report assay strategy on murine spermatocyte cell line GC-2, we confirmed the physical interaction among circLIMA1 and two miRNAs, miR-7a-5p and miR-214-3p; then, by miRNA mimics transfection experiments, we demonstrated that such an interaction is involved in the cytoskeletal regulation of actin, unbalancing the relative levels of RNA molecules by increasing the miRNAs level with miRNA transfection. In this scenario, we tried to unveil circLIMA1 role in the dynamic actin remodeling in SPZ during their epididymal transit. Interestingly, a differential content of circLIMA1, closely confined to sperm head, was shown along the epididymis. In order to shed light on a peculiar circRNA-dependent mechanism in sperm nucleus, differently from what was suggested in GC-2, we hypothesized that the RBP QKI - able to physically interact with circLIMA1 - may act as a shuttle to drive circLIMA1 nuclear localization and the nuclear organization of actin, as a consequence.

In conclusion the current work provides new insights into: *i*) the endocannabinoid contribution to spermatic circRNA cargo, *ii*) the active role of circRNAs in the regulation of actin filament dynamism during epididymal transit, *iii*) a new intriguing molecular interaction between QKI and circLIMA1 involved in sperm nucleus architecture.

## Material and Methods

### Experimental animals

Mice (*Mus musculus*) genetically deleted for *Cb1* were provided by Prof. Ledent 25. Male and female CB1 heterozygous (CB1^+/-^) mice have been maintained on a CD1 background (Charles River Laboratories, Lecco, Italy) to expand colony, then used to generate adult WT, CB1^+/-^ and CB1^-/-^ male mice. All the animals were kept in a room with controlled temperature (22 ± 2°C), ventilation, and lighting (12-h light/dark cycles) with a standard pellet diet and free access to water.

Adult males (3-5 months) under anaesthesia were sacrificed by cardiac perfusion with PBS (pH 7.6) to clean peripheral tissues (testes, epididymides and SPZ) from blood contaminants. In detail, testes were rapidly removed and stored at -80°C, while epididymides were dissected and used to collect total epididymal SPZ or SPZ from *caput* (*caput* SPZ) and *cauda* (*cauda* SPZ) regions, and related epididymal tissues (SPZ-deprived epididymis), depending on the experimental procedure, as described below.

The number of the enrolled adult animals was determined by the parameters established through the G*Power analysis (latest ver. 3.1.9.7; Heinrich-Heine-Universität Düsseldorf, Düsseldorf, Germany; http://www.gpower.hhu.de/) required to get the permission for *in vivo* experiments in Italy, suggested by the Legal Entity giving the permission.

Experiments involving animals were approved by the Italian Ministry of Education and the Italian Ministry of Health, with authorization n°941/2016-PR issued on 10.10.2016. Procedure involving animal care were carried out in accordance with the National Research Council's publication Guide for Care and Use of Laboratory Animals (National of Institutes of Health Guide).

### Mouse sperm collection from caput and cauda epididymis

Total epididymis (from n=6 WT and n=6 CB1^-/-^) or *caput/cauda* epididymis (from n=3 WT) were separately immersed in PBS (pH 7.6) and cut to let SPZ flow out from the ducts. Samples of total and/or *caput* and *cauda* SPZ were then filtered throughout cheesecloth to eliminate fragments of epididymal tissue and centrifuged at 1500 x *g* for 30 min at 4°C. The epididymal fragments were separately frozen as pieces of total or *caput* and *cauda* epididymis deprived of sperm cells.

After centrifugation, the SPZ pellet was incubated on ice for 30 min with Somatic Cell Lysis Buffer (SCLB; 0.1% SDS, 0.5% Triton X-100 in DEPC-H_2_O) to eliminate possible contamination by somatic cells. Microscope examination was carried out to verify the optimal elimination of somatic cells. After lysis, SPZ were centrifuged at 800 x *g* for 15 min at 4°C and then washed twice with PBS. Aliquots of total epididymal SPZ from WT and CB1^-/-^ or *caput* and *cauda* SPZ from WT mice, were both stored at -80°C for RNA or protein extraction and dried on slides to be finally stored at -20°C for immunofluorescence analysis.

### Total RNA preparation

Total RNA was extracted from murine tissues, SPZ and GC-2 cells using Trizol Reagent (Invitrogen Life Technologies, Paisley, UK) following the manufacturer's instructions. In brief, samples were homogenized in Trizol Reagent (1 ml Trizol Reagent/mg tissue or 5-10 x 10^6^ sperm cells); after homogenization, samples were incubated for 5 min at 20°C to allow the complete dissociation of nucleoprotein complexes. Then 0.2 ml chloroform/ml Trizol Reagent were added and the sample centrifuged at 12000 x *g* for 15 min at 4°C. The aqueous phase was transferred to a fresh tube and total RNA was precipitated by mixing with isopropyl alcohol (0.5 ml/ml Trizol Reagent) and 1 µl glycogen (20 mg/ml) to promote the precipitation of small size RNAs. After centrifugation at 12000 x *g* for 10 min at 4°C, the RNA pellet was washed with 75% ethanol, centrifuged at 7500 x *g* for 10 min at 4°C and dissolved in an appropriate volume of DEPC-treated water. The quantity (ng/µl) and purity (260/280 and 260/230 ratios) of total RNAs were assessed with a NanoDrop 2000 spectrophotometer (Thermo, Waltham, MA, United States). To remove potential contamination of genomic DNA, RNA aliquots (10 µg) were treated with 2U DNase I (RNase-free DNase I, Ambion, Thermo Fisher Scientific, Massachusetts, United States). The RNAs were then preserved at -80°C until the next step.

### CircRNA microarray

The sample preparation and microarray hybridization were performed according to the Arraystar's standard protocols (Arraystar, Rockville, MD). Briefly, the enriched circRNAs were amplified and transcribed into fluorescent cRNA utilizing a random priming method according to Arraystar Super RNA Labeling protocol (Arraystar, Inc.). The labeled cRNAs were purified by RNeasy Mini Kit (Qiagen, Germantown, Maryland, USA). The concentration and specific activity of the labeled cRNAs (pmol Cy3/μg cRNA) were measured by NanoDrop ND-1000. One μg of each labeled cRNA was fragmented by adding 5 μl 10 × Blocking Agent and 1 μl of 25 × Fragmentation Buffer, then heated the mixture at 60°C for 30 min, finally 25 μl 2 × Hybridization buffer was added to dilute the labeled cRNA. Fifty μl of hybridization solution were dispensed into the gasket slide and assembled to the circRNA expression microarray slide. The slides were incubated for 17 hours at 65°C in an Agilent Hybridization Oven. The hybridized arrays were washed, fixed and scanned using the Agilent Scanner G2505C (Agilent Technologies, CA).

### Expression Profiling Data

Agilent Feature Extraction software (version 11.0.1.1) was adopted to analyze acquired array images. Quantile normalization of raw data and subsequent data processing were performed using the R software package (version 4.2 Limma, Bioconductor). After quantile normalization of the raw data, low intensity filtering was performed, and the circRNAs that at least 3 out of 6 samples have flags in “P” or “M” (“All Targets Value”) were retained for further analyses.

### Differential expression analysis

Two groups of circRNAs were identified in WT and in CB1^-/-^ SPZ, respectively, by using the R software package and conveniently compared by t-test in order to select DE-circRNAs, with p-value<0.05 and fold change>1.5. Since the comparison between WT and in CB1^-/-^ SPZ, we used along the text the expression “CB1^-/-^ compared to WT SPZ” to indicate statistically significant DE-circRNAs as showed by volcano plot filtering. DE-circRNAs among samples were identified through Fold Change filtering. Hierarchical clustering was performed to show the distinguishable circRNA expression pattern among samples.

### Functional Annotation for circRNA/miRNA and target miRNA Interaction

The circRNA/miRNA interaction was predicted with Arraystar's miRNA target prediction software based on both TargetScan 26 and MiRanda online analytical software 27; such an analysis was performed for all DE-circRNAs.

For functional annotation, all parental genes of the DE-circRNAs were subjected to Gene Ontology (GO) enrichment and Kyoto Encyclopedia of Genes and Genomes (KEGG) (www.genome.jp/kegg) pathway enrichment analyses using DAVID Bioinformatics Resources 6.8 (david.ncifcrf.gov/home.jsp). The p-value was calculated using a hypergeometric test and corrected by Benjamini-Hochberg adjustment. We regarded the Fold Enrichment as the enrichment score that indicated the significance of correlation. Validated or predicted targets of miRNAs were retrieved by Diana TarBase 8.0 (http://www.microrna.gr/tarbase); circRNA/miRNA/Target network was built and visualized by using Bisogenet plug-in of Cytoscape (www.cytoscape.org).

### Reporter constructs

The circLIMA1 sequences potentially targeted by miRNAs were chemically synthesized and cloned into psiCheck‐2 vector (Promega, Madison, WI, USA). In detail, couples of oligonucleotides, representing the target sites for the selected miRNAs, and carrying additional upstream *Xho*I and *Eco*RV restriction sites and a downstream *Not*I site, were annealed and ligated into *Xho*I and *Not*I sites of the psiCheck‐2 vector; *Eco*RV digestions and sequencing were then used to identify recombinant clones. Control plasmids (indicated as I) for miRNAs target sequence were obtained by the same approach, with the exception that the cloned couple of oligonucleotides represented the inverted target sequence.

### Cell culture, transfections and luciferase and qPCR miRNA assays

Murine spermatocyte cell line GC-2 was cultured in DMEM containing 10% fetal bovine serum, 2mM L‐glutamine, 50 U/ml penicillin, and 100 mg/ml streptomycin (Sigma-Aldrich, Milano, Italy). The day before transfection, the cells were trypsinized and seeded in a medium without antibiotics in 12‐well plates. Cells at 80-90% of confluence were transfected with: 50 nM miRIDIAN microRNA mmu-miR-7a-5p mimic or mmu-miR-214 (Dharmacon - Horizon Discovery, Cambridge, UK) along with 0.2 μg of reporter constructs for the luciferase assays, or alone for phalloidin staining experiments, by using 3 μl of Lipofectamine 2000 (Invitrogen) for 1 μg of nucleic acids, as described by the manufacturer. Luciferase assays were performed 48 hours after transfection by using the Dual‐Luciferase Reporter Assay System (Promega, Madison, WI, USA) according to the manufacturer's protocol. All the analyses were performed on three independent experiments, each in triplicate. Comparison of data sets in the different experiments was performed by Student's t test and a value of p<0.05 was considered statistically significant. MiR-214, miR-7a-5p and the reference U6 snRNA were quantified by miRCURY LNA miRNA SYBR^®^ Green PCR System (Qiagen, Germantown, Maryland, USA) according to the manufacturer's protocol. The expression levels of miRNAs were normalized to U6 by using the 2^-∆Ct^ method and reported as fold change in comparison to control experiments. Phalloidin staining was performed as following described.

### RNA expression analysis by One-Step Evagreen qRT-PCR

We investigated circRNA expression through One-Step Evagreen qRT-PCR reaction by using a kit containing qRT-PCR enzyme mix and an Evagreen qPCR Mastermix (Applied Biological Materials Inc., Ferndale, WA, United States), according to the manufacturer's instructions. All reactions were performed using 50 ng of total RNA on a CFX-96 Real Time PCR System (Biorad, Milano, Italy). Assays were carried out in triplicates and included a melting curve analysis for which all samples displayed single peaks for each primer pairs. A negative control, without RNA, was also included. RNA expression was evaluated through CFX Manager software (Biorad, Milano, Italy). Normalization was performed using *Cyclophilin* and *RPS18* as housekeeping genes for SPZ and tissues, respectively. For validation of circRNAs, normalized fold expression (nfe) of circRNAs was calculated by applying the 2^-∆∆Ct^ method, while for circLIMA1, data analysis was expressed by calculating delta-Cq values with the aim of expressing the pure expression profile. All the results were expressed as mean value of nfe ± SEM.

### Vesicle shuttle *in vitro* experiment and Cytochalasin-D treatment

Vesicle shuttle experiments were carried out as previously reported 6. In brief, *caput* epididymis from WT (n=5) and CB1^-/-^ (n=5) mice were separately pulled in PBS (pH 7.6) and cut to let SPZ flow out from the ducts. Then, *caput* SPZ samples were filtered throughout cheesecloth to eliminate fragments of epididymal tissue and centrifuged at 1500 × *g* for 30 min at 4°C. The resulting *caput* fluid was further clarified *via* centrifugation (16000 × *g* for 30 min at 4°C) with the supernatant yielding the ELF. The resulting *caput* SPZ pellet and *caput* ELF were used for *in vitro* treatments as follows.

For each experimental group, 10 × 10^6^ of WT SPZ from *caput* epididymis were incubated for 15 min at 37°C in 1 ml of: 1) WT *Caput* ELF (CTRL group); 2) WT *Caput* ELF combined with Cytochalasin-D (C8273; Sigma-Aldrich, Milano, Italy) at the concentration of 10 μM; 3) CB1^-/-^ *Caput* ELF; 4) CB1^-/-^ *Caput* ELF combined with Cytochalasin-D 10 μM. After treatment, SPZ were centrifuged at 1500 ×* g* for 20 min at 4°C and washed twice with PBS. Sperm pellets were stored at -80°C for RNA extraction and dried on slides to be finally stored at -20°C for immunofluorescence analysis.

### Immunofluorescence analysis

GC-2 cells,* caput* and* cauda* SPZ from WT (n=3) and CB1^-/-^ (n=3) mice and WT SPZ from *caput* epididymis *in vitro* treated as above described, dried on slides as above reported, were fixed in 4% paraformaldehyde (sc-281692; Santa Cruz Biotechnology, Heidelberg, Germany) for 20 min at RT and then permeabilized with 0.1% Triton X-100 (X100; Sigma-Aldrich, Milano, Italy). After permeabilization, phalloidin staining (49409; Sigma-Aldrich, Milano, Italy) was carried out for 1 hour at 37°C. Following three washes in Dulbecco's PBS (DPBS, 1X), nuclei were labeled with DAPI (D9542; Sigma-Aldrich, Milano, Italy) and the analysis was conducted under an optical microscope (Leica DM 5000 B + CTR 5000) with a UV lamp. For QKI immunofluorescence analysis, *caput* and* cauda* SPZ from WT (n=3) and CB1^-/-^ (n=3) mice were fixed and permeabilized as above reported. Following permeabilization step, blocking was conducted with 10% of donkey serum (ab7475; Abcam, Cambridge, UK) for 30 min and the cells were then incubated with QKI Ab (PA5-87292; Invitrogen, Milano, Italy), ON at 4°C. Following three washes in Dulbecco's PBS (DPBS, 1X), a fluorescein isothiocyanate (FITC) conjugated Ab was used (711-095-152; Jackson ImmunoResearch, Cambridge, UK) for 1 hour at 37°C. Then phalloidin staining was carried out for 1 hour at 37°C and nuclei were labeled with DAPI. The analysis was conducted under an optical microscope (Leica DM 5000 B + CTR 5000) with a UV lamp.

### Sonication-Resistant-sperm Nuclei (SRN) preparation

Sperm nuclei isolation was performed as reported by Cacciola et al. 20. In brief, *caput* and *cauda* SPZ (from n=3 WT and n=3 CB1^-/-^) were collected as above reported. Then, SPZ were resuspended in TNE buffer (0.1 M Tris-HCl pH7.6, 0.15 M NaCl, 1 mM EDTA pH 8) containing protease inhibitors (10 μg/ml of leupeptin, aprotinin, pepstatin A, chymostatin, and 5 μg/ml of TPCK) and sonicated on ice for 1 burst 15 sec with a mild 40% output power. SRN were separated from debris in 50% sucrose cushion (1 ml) by centrifugation at 8000 × g for 60 min at 4°C, washed twice in TNE buffer and used for RIP experiments, as following described.

### RNA binding protein immunoprecipitation assay (RIP)

For RIP assay, 1 × 10^7^ of WT and CB1^-/-^ SRN collected from *caput* and *cauda* epididymis were lysed in 500 µl of RIP lysis buffer (50 mM Tris-HCl pH 7.4; 150 mM NaCl; 5 mM EDTA; 1% NP-40; 0.1% SDS) supplemented with protease inhibitors (10 μg/ml of leupeptin, aprotinin, pepstatin A, chymostatin, and 5 μg/ml of TPCK) and RNase inhibitors (100 U/ml). An aliquot of total lysate was removed from each sample for following input analysis. Equal concentration of lysate was incubated with 5 µg of QKI Ab (PA5-87292; Invitrogen, Milano, Italy) or IgG (12370; Sigma-Aldrich, Milano, Italy) under rotary agitation at 4°C ON. Afterwards 60 µl of slurry of Protein A/G PLUS Agarose Beads (sc-2003; Santa Cruz Biotechnology, Heidelberg, Germany) was added to each sample and incubated at 4°C for 4 hours. Then pellets were washed four times with cold TBS pH 7.6 at 3000 × g for 5 min at 4°C. An aliquot consisting of 10% of total beads was removed before RNA isolation from each sample for the following immunoprecipitated protein analysis by western blot. After washes, pellets of beads were resuspended in 500 µl of Trizol Reagent (Invitrogen Life Technologies, Paisley, UK) and RNAs were eluted following the manufacturer's instructions. The immunoprecipitated RNAs with QKI and IgG control were quantized (ng/µl) using a NanoDrop 2000 spectrophotometer (Thermo, Waltham, MA, United States) and used for circLIMA1 qRT-PCR analysis.

### Western blot analysis

Total lysate of WT and CB1^-/-^ SRN collected from *caput* and *cauda* and aliquots of beads removed during RIP experiments before RNA isolation, were separated by SDS-PAGE and transferred to polyvinylidene difluoride membrane (GE Healthcare, Milano, Italy) at 280 mA for 2.5 hours at 4°C. The filters were treated for 3 hours with blocking solution [5% nonfat milk, 0.25% Tween-20 in Tris-buffered saline (TBS, pH 7.6)] and then separately incubated ON, at 4°C in TBS-milk buffer (TBS pH 7.6, 3% nonfat milk) with different primary antibodies [QKI (PA5-87292), Invitrogen, Milano, Italy, diluted 1:500; Gelsolin (sc-514502), Santa Cruz Biotechnology, Heidelberg, Germany, diluted 1:500]. After washing in 0.25% Tween20-TBS, filters were incubated with 1:1000 horseradish peroxidase-conjugated rabbit IgG (Dako Corp., Milano, Italy) in TBS-milk buffer and then washed again. The immune complexes were detected using the enhanced chemiluminescence-western blotting detection system [Amersham ECL western Blotting Detection Reagent (RPN2106) GE Healthcare, Milano, Italy]. The specificity of the immunoreactions was routinely checked by omitting primary Ab (data not shown).

### PCR primer design

Primers to validate and amplify selected circRNAs in murine SPZ samples were designed through the online tool Primer-BLAST (http://www.ncbi.nlm.nih.gov/tools/primer-blast/). In order to make primers specific for the circular isoforms, we designed primers spanning the back-splicing junction. We also designed specific primers for the housekeeping gene used for normalization: *Cyclophilin* and *Ribosomal Protein S18* (*RPS18*) for SPZ and tissues, respectively. Primer sequences are shown in Table **1**.

### Statistical Analysis

ANOVA followed by Student's t-test and Duncan's test (for multi group comparison), was used to identify groups having different mean. Differences with p<0.05 were considered statistically significant. Data were expressed as the mean ± SEM from at least 3 independent animals for each genotype or experimental group. For qRT-PCR analyses, triplicates from each of 3 animals/genotype or experimental group were considered.

## Results

### Overview of circRNA expression in WT and CB1^-/-^ epididymal SPZ

The expression profile of circRNAs in epididymal SPZ from WT (n=3) and CB1^-/-^ (n=3) mice was analyzed using microarray technology.

**Figure 1:**
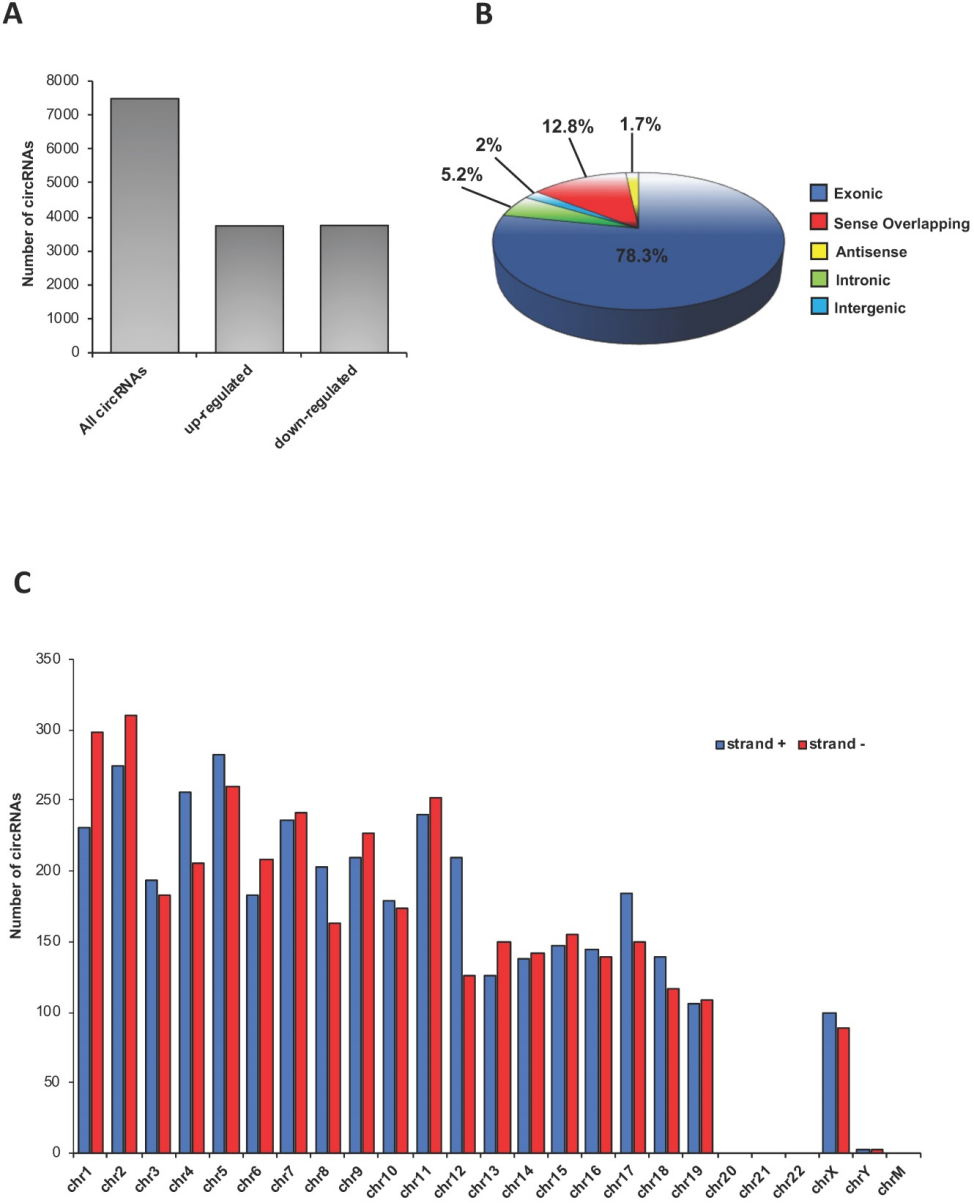
** Overview of circRNA expression in mouse SPZ**. (**A**) The distribution of up- and down-regulated circRNAs in CB1^-/-^ (n=3) compared to WT (n=3) SPZ among a total of 7486 circRNAs. (**B**) The proportion of different types of circRNAs among all predicted circRNAs. (**C**) Chromosomal distribution of SPZ derived circRNAs, on strand + and strand -, according to their host gene location.

A total of 7486 circRNAs was identified in epididymal SPZ; roughly an equal number appeared up- (n=3736) or down-regulated (n=3750) in CB1^-/-^ compared to WT SPZ (Figure 1**A**). According to their structure, circRNAs were distinguished in exonic, intronic, intergenic, sense overlapping and antisense. Exonic circRNAs (78.3%) were the most abundant type of circRNAs in SPZ, while intergenic (2%) and antisense (1.7%) were less represented (Figure 1**B**). According to the location of their host genes, circRNAs were widely distributed across almost all chromosomes (Figure 1**C**). However, the highest number of circRNAs was produced on chromosomes 1, 2 and 5, while the lowest one was generated by chromosomes 19, X and Y. No circRNAs were produced on chromosomes 20, 21, 22 and mitochondrial (M). Furthermore, circRNAs distributed on chromosomes 4, 5, 8, 12, 17 and 18 predominantly derived from strand +, while those distributed on chromosomes 1, 2, 6, 9, 11 and 13 showed a reversed profile. Finally, circRNAs distributed on the remaining chromosomes derived equally from the strand + and - (Figure 1**C**).

### Differential expression of circRNAs in CB1^-/-^ *vs* WT epididymal SPZ

**Figure 2:**
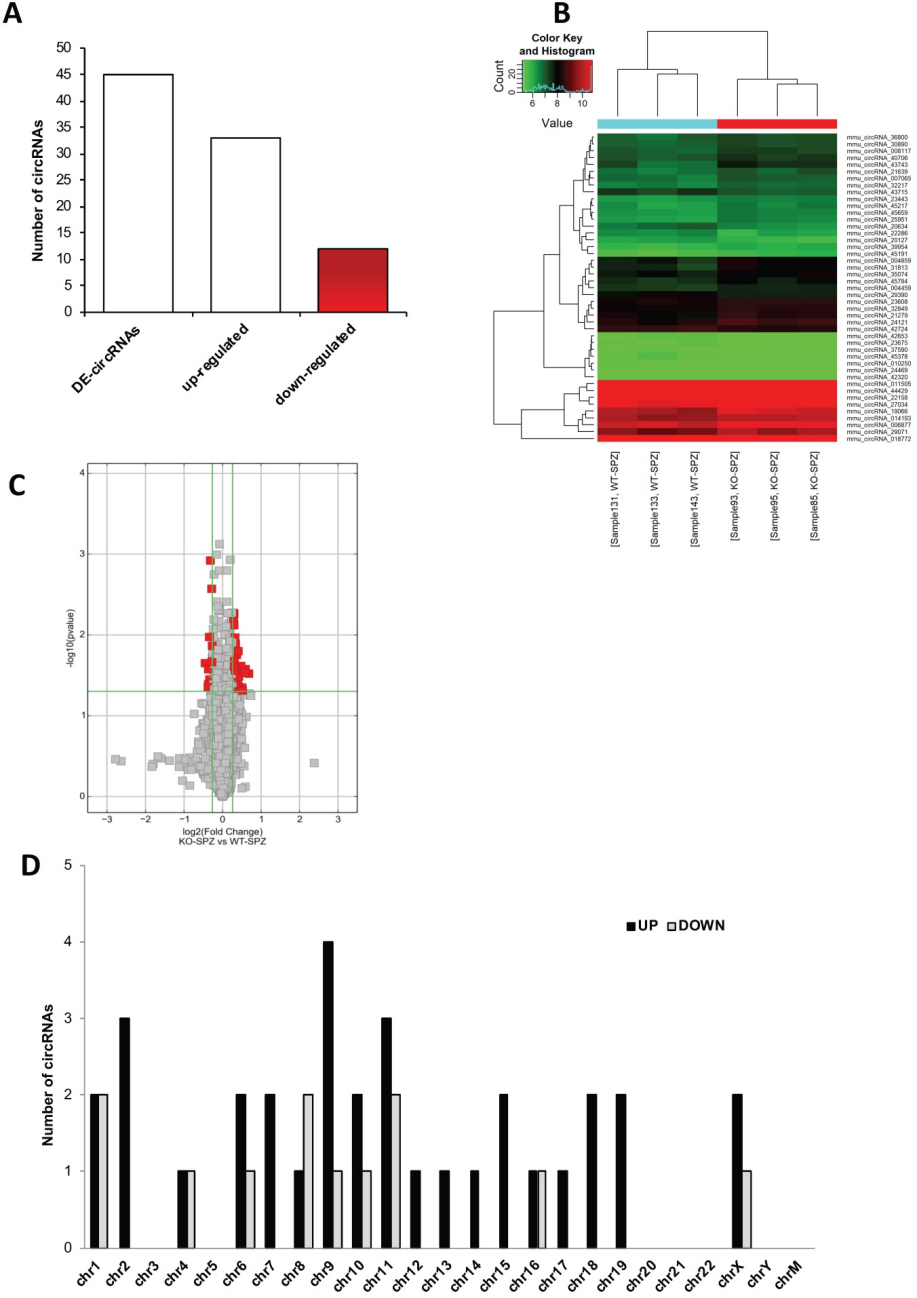
**Differential expression of circRNAs between WT and CB1^-/-^ SPZ**. (**A**) The distribution of up- and down-regulated DE-circRNAs, among a total of 45 DE-circRNAs, in CB1^-/-^ compared to WT SPZ. (**B**) Hierarchical clustering analysis of DE-circRNAs in WT SPZ (samples 133, 143, 131) and CB1^-/-^ SPZ (samples 85, 95, 93); the expression values (Fold change>1.5, p<0.05) were represented in different colors, indicating expression levels above and below the median expression level across all samples. (**C**) The volcano plot was constructed using Fold-Change and p-values; in detail, the values on X and Y axes are log2 (FC = Fold-Change) and -log10 (p-values), respectively. Red points in the volcano plot represent the DE-circRNAs with statistical significance. (**D**) The distribution of up- and down-regulated DE-circRNAs in the mouse genome, according to their host gene location.

A finer circRNA microarray analysis, using more stringent parameters such as fold change cut-off >1.2 and p-values cut-off <0.05, identified a total of 45 differentially expressed (DE)-circRNAs, including 33 up-regulated and 12 down-regulated in CB1^-/-^ compared to WT SPZ (Figure 2**A**).

The DE-circRNAs ranked by fold change, including both up- and down-regulated, were presented as a heatmap (Figure 2**B**). Volcano plot analysis was used to assess the variations in circRNA expression profiles between the two groups (Figure 2**C**).

According to their host gene location, the distribution of DE-circRNAs was analyzed in mouse genome (Figure 2**D**); interestingly, chromosomes 1, 4, and 16 contained an equal number of both up- and down-regulated DE-circRNAs in CB1^-/-^ compared to WT SPZ. Furthermore, chromosomes 6, 9, 10, 11, 16 and X especially contained up-regulated circRNAs in CB1^-/-^ compared to WT SPZ; this profile was reversed only on chromosome 8. No DE-circRNA was detected on chromosomes 3, 5, 20-22, Y and M; instead all remaining chromosomes contained only up-regulated circRNAs in CB1^-/-^ compared to WT SPZ (Figure 2**D**).

### Functional Clustering, Gene Ontology and KEGG analysis of DE-circRNAs

DE-circRNAs were selected to predict the association with miRNAs. According to the results of bioinformatic prediction, different functional ceRNETs were built (Supplementary Figure S1).

In Figure S1**A**, two circRNAs (ovoid symbols) - whose host genes are members of the same gene family but are located on different chromosomes - showed tethering activity toward the same miRNA (rectangular symbol). Interestingly, some circRNAs - whose host genes are located on different chromosomes - can tether both the same miRNA (Figure S1**B**) and different miRNA isoforms (Figure S1**C**). The cases above described were representative of circRNAs up-regulated in CB1^-/-^ compared to WT SPZ.

The results of Gene Ontology (GO) functional enrichment analysis indicated that both up- and down-regulated DE-circRNAs in CB1^-/-^ SPZ were involved in several biological processes, including regulation of actin filament organization, actin filament bundle assembly and actin filament-based movement. The top 40 results with the most significant p-values of up- and down-regulated DE-circRNAs in CB1^-/-^ SPZ were presented in the bar blot (Supplementary Figure S2**A** and **B**, respectively).

KEGG pathway analysis was then performed for the target genes of SPZ-derived circRNAs; the top 20 enriched KEGG signaling pathways are shown in Supplementary Figures S3**A** and S3**B**. Interestingly, the KEGG pathways associated with up-regulated circRNAs in CB1^-/-^ SPZ were linked to VEGF signaling, Notch signaling, GnRH signaling, oocyte meiosis, endocytosis and cell cycle (Figure S3**A**). Conversely, the KEGG pathways associated with down-regulated circRNAs in CB1^-/-^ SPZ were linked to mRNA surveillance, ubiquitin-mediated proteolysis, adherens junctions, inositol phosphate metabolism, phosphatidylinositol signaling (Figure S3**B**).

### Experimental Validation of Predicted circRNAs in mouse SPZ, testis and epididymis

Experimental validation of circRNA microarray results was carried out. A quality control of RNA extracted from SPZ was considered as previously described 12. Briefly, in all samples, we analyzed the expression of non-sperm cell markers, such as CD4 (biomarker of leukocytes) and E-cadherin (biomarker of epithelial cells) to exclude some possible contamination by somatic cells (i.e., blood and epididymal cells). No significant signal was amplified by qPCR (data not shown).

For the validation by qPCR, we decided to choose those DE-circRNAs - predicted in mouse SPZ and identified by circRNA microarray - that showed a significant high score of normalized intensity and whose host genes were related to sperm physiology and functions.

**Figure 3:**
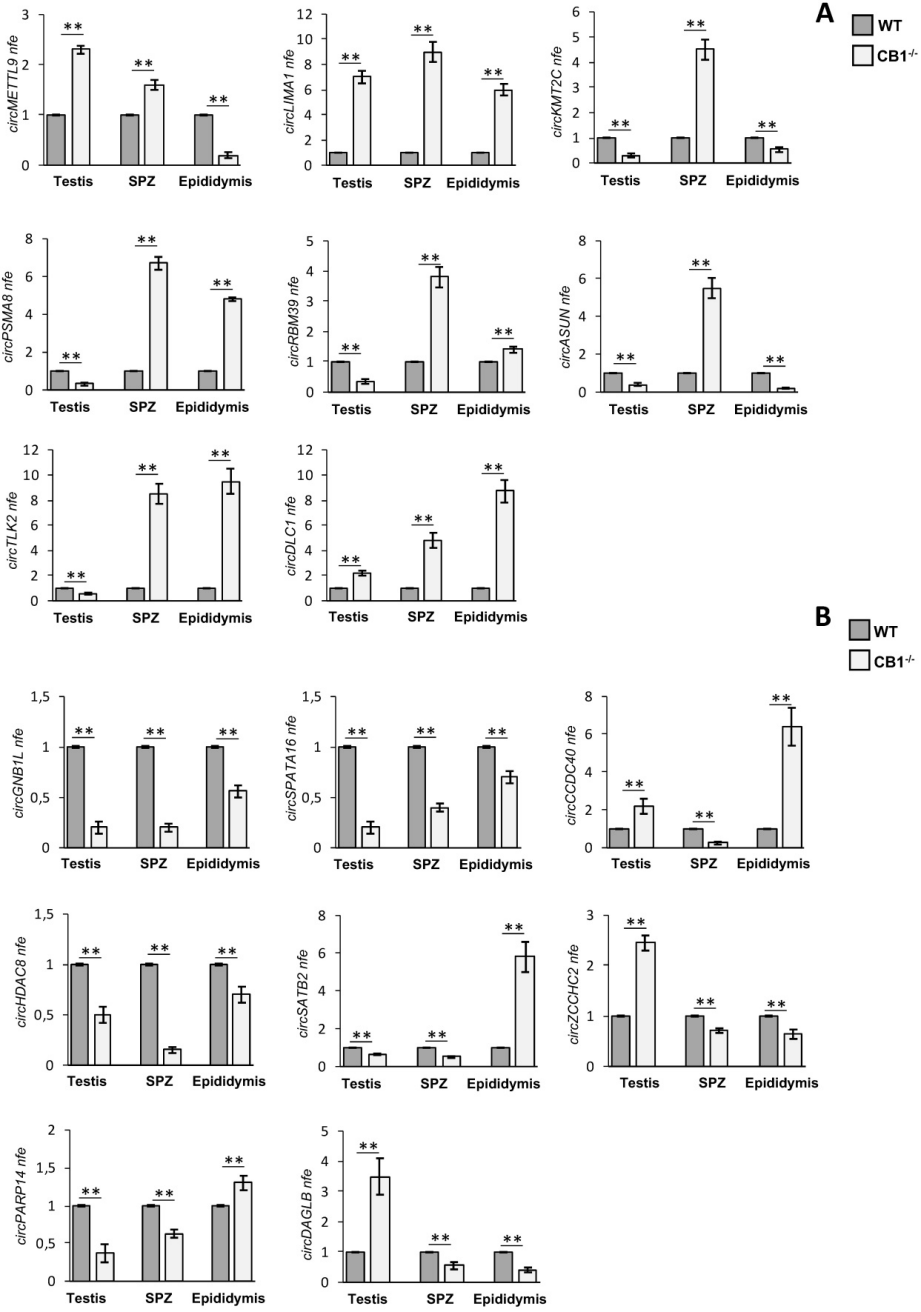
** Validation of circRNA microarray results in testis, SPZ and epididymis from WT and CB1^-/-^ mice**. (**A**) Expression analysis of 8 circRNAs up-regulated in CB1^-/-^ compared to WT derived SPZ. (**B**) Expression analysis of 8 circRNAs down-regulated in CB1^-/-^ compared to WT derived SPZ. qRT-PCR data are normalized using *Cyclophilin* and *RPS18*, for SPZ and tissues respectively, expressed as fold expression (nfe) and reported as mean value ± S.E.M.**: p< 0.01.

Thus, we searched relative sequences in circBase (http://www.circbase.org) and designed specific primers for circular isoforms, spanning the back-splicing junction to use for One-Step Evagreen qRT-PCR analysis (Figures 3**A** and **B**). The quality of melting curves for each primer pair was carefully checked and only curves with single peaks were considered suitable for further analysis (data not shown).

Furthermore, to understand if the derivation of the sperm circRNA content could be of testicular or epididymal origin, we evaluated the expression of the related circRNAs in the SPZ, as well as in the testis and epididymis.

QPCR analysis performed in CB1^-/-^ and WT epididymal SPZ showed the expression of circRNAs up-regulated (p<0.01; Figure 3**A**) and down-regulated (p<0.01; Figure 3**B**) in CB1^-/-^ compared to WT SPZ, respectively, thus, confirming circRNA microarray results. Interestingly, some circRNAs showed in the testis and the epididymis the same expression profile validated in SPZ, remaining up-regulated (circLIMA1, circDLC1, Figure 3**A**) or down-regulated (circGNB1L, circSPATA16, circHDAC8, Figure 3**B**), respectively; others, appeared with an opposite trend to that observed in SPZ (i.e. circKMT2C, circASUN in Figure 3**A** and circCCDC40 in Figure 3**B**).

Intriguingly, circRNAs up- or down-regulated in SPZ and epididymis, but down- or up-regulated in testis, respectively (circPSMA8, circRBM39, circTLK2 in Figure 3**A**, circZCCHC2 and circDAGLB in Figure 3**B**) were observed, indicating probably, an epididymal contribution for these spermatic circRNAs. Conversely, we noted an up-regulated circRNA (circMETTL9, Figure 3**A**) and two down-regulated circRNAs (circSATB2 and circPARP14 in Figure 3**B**) with an equal expression profile in SPZ and testis and opposite profile in epididymis, likely suggesting their testicular origin.

### CircLIMA1-dependent ceRNET is involved in the cytoskeletal regulation of actin

**Figure 4:**
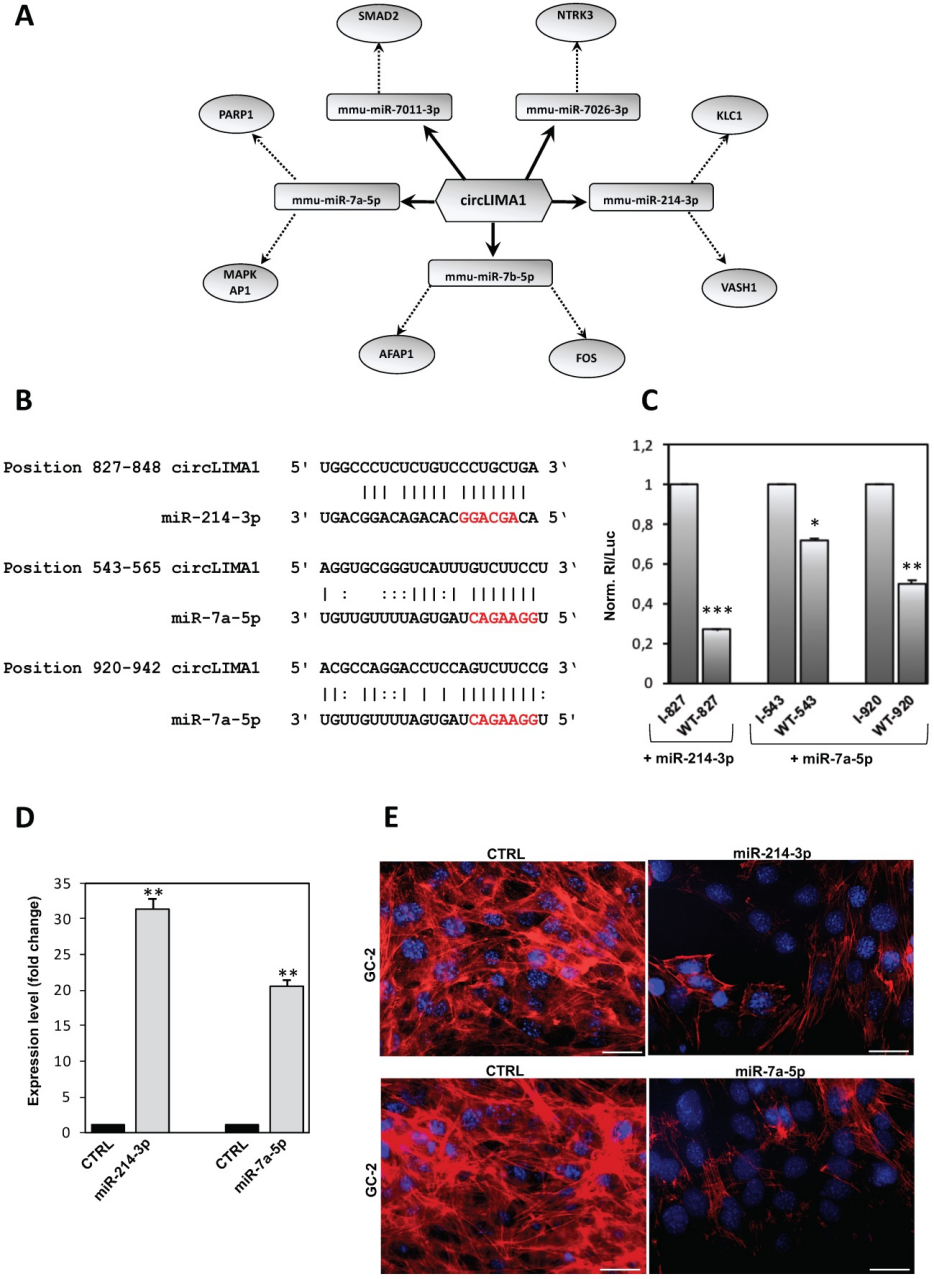
** CircLIMA1-dependent ceRNET validation and its involvement in the regulation of cytoskeletal actin.** (**A**) Analysis of circLIMA1*,* up-regulated in CB1^-/-^ compared to WT derived SPZ that tethers a group of miRNAs as targets, all involved in cytoskeleton-dependent pathways. Networks were built using Cytoscape. Hexagonal and rectangular symbols represent circRNAs and miRNAs, respectively. The arrow indicates the tethering activity of circRNAs toward miRNAs, while the dotted arrow indicates the pathways upstream of the miRNAs. (**B**) Prediction of miR-214-3p and miR-7a-5p binding site on circLIMA1 sequence; miRNA seed sequences are marked in red. (**C**) GC-2 cells were transfected with the luciferase-based reporter constructs containing the wild-type target sequence of miRNA (WT-starting nucleotide position number) or the control plasmids with inverted target sequence (I- starting nucleotide position number), along with 50 nM of the indicated miRNA mimic. After 48 hours, luciferase activities were recorded; the Renilla luciferase activity (Rl) was normalized to the firefly luciferase activity (Luc), whose coding sequence is carried by the same vectors; the values are reported as fold mean ± S.E.M. relative to RL/Luc recorded for transfection of controls (specific I construct + miRNA mimic) which were set to 1. Data are the mean ± S.E.M of three independent experiments, each with three data sets. *:p<0.05, **:p<0.01, ***:p<0.001. (**D**) qRT-PCR expression analysis of miR-214-3p and miR-7a-5p in GC-2 cells after 48 hours of miRNA mimic transfection. All qRT-PCR data are normalized using U6, expressed as fold change and reported as mean value ± S.E.M. ***: p<0.001. (**E**) Immunofluorescence analysis of F-actin by phalloidin staining (red) in GC-2 cells transfected with 50 nM of the indicated miRNA mimic. (n=3 different samples for each experimental group). Nuclei were labeled with DAPI (blue). Scale bar: 50 µm.

Considering that circRNAs are able to harbor several miRNAs 28, the construction of a ceRNET is useful to shed light on predicted mRNA targets. Among circRNAs up-regulated in CB1^-/-^ SPZ, we were interested in circLIMA1 since its linear transcript encodes for an actin-binding protein involved in actin cytoskeleton regulation and dynamics, inhibiting actin filament depolymerization and cross-linking filaments in bundles 24. According to the results of the bioinformatic prediction derived from microarray analysis, five miRNAs were identified: mmu-miR-7011-3p, mmu-miR-7026-3p, mmu-miR-214-3p, mmu-miR-7b-5p, and mmu-miR-7a-5p (Figure 4**A**). The mRNAs targets were preferentially involved in cytoskeleton-dependent pathways, as discussed below.

Within the identified miRNAs predicted to bind to circLIMA1 sequences, we focused our attention on those miRNAs classified as “with confidence” (experimentally validated) and/or found as “>100 reads” on miRBase database, thus selecting miR-7a-5p and miR-214-3p. Reasoning that RNA regulatory networks occur if the involved molecules are co-expressed, we measured the expression of selected miRNAs in WT and CB1^-/-^ SPZ and tissues (testis and epididymis), thus finding miR-7a-5p and miR-214-3p expressed in the analyzed samples (data not shown).

MiR-214-3p and miR-7a-5p have one and two predicted binding sites on circLIMA1, respectively (Figure 4**B**). Those target sites were subjected to a validation test based on luciferase reporter constructs transfected in GC-2 cell line. In detail, circLIMA1 sequences predicted as miRNA target sites (sequence 827-848 targeted by miR-214-3p; sequences 543-565 and 920-942 targeted by miR-7a-5p) were singularly cloned downstream the Renilla reniformis luciferase (Rl) coding sequence carried by the psi‐Check‐2 vector. The obtained reporter constructs were transfected in GC-2 cells along with mmu-miR-214-3p or mmu-miR-7a-5p miRNA mimics and luciferase activity was evaluated. The expected results were the observation of a reduction in the luciferase activity if a given miRNA binds to the circLIMA1 target sequence, in comparison to the activity of a control, represented by the reporter vector carrying the inverted target sequence. As showed, the sequence 827-848, potentially targeted by miR-214-3p, drastically inhibited the expression of the reporter construct transfected along with the miRNA mimic, with a registered luciferase activity approximately 73% lower (p<0.001) than the control reporter vector (indicated as I) (Figure 4**C**). Similarly, the sequences 543-565 and 920-942 significantly reduced the expression of the reporter construct transfected along with miR-7a-5p mimic, showed a luciferase activity approximately 30% and 50% lower than the controls (p<0.05; p<0.01), respectively (Figure 4**C**), confirming that both miR-214-3p and miR-7a-5p were able to directly bind to circLIMA1.

In order to demonstrate the involvement of miR-214-3p and miR-7a-5p in the cytoskeletal regulation of actin, GC-2 cells were transfected with mmu-miR-214-3p or mmu-miR-7a-5p miRNA mimics and F-actin was analyzed by immunofluorescence analysis through phalloidin staining. Expression analysis carried out by qRT-PCR, confirmed significant higher levels of miR-214-3p (p<0.01) and miR-7a-5p (p<0.001) than CTRL groups, following miRNA mimics transfection (Figure 4**D**). Phalloidin staining for F-actin evaluation was compared between CTRL and transfected cells with mmu-miR-214 or mmu-miR-7a-5p miRNA mimics. As showed, following miRNA mimics transfection, a dramatic reduction of F-actin signal occurred in association with a complete deregulation of actin-cytoskeleton architecture (Figure 4**E**), confirming the active role of miR-214-3p and miR-7a-5p in the cytoskeletal organization of actin.

### CircLIMA1-dependent regulation of the sperm nuclear actin organization

**Figure 5:**
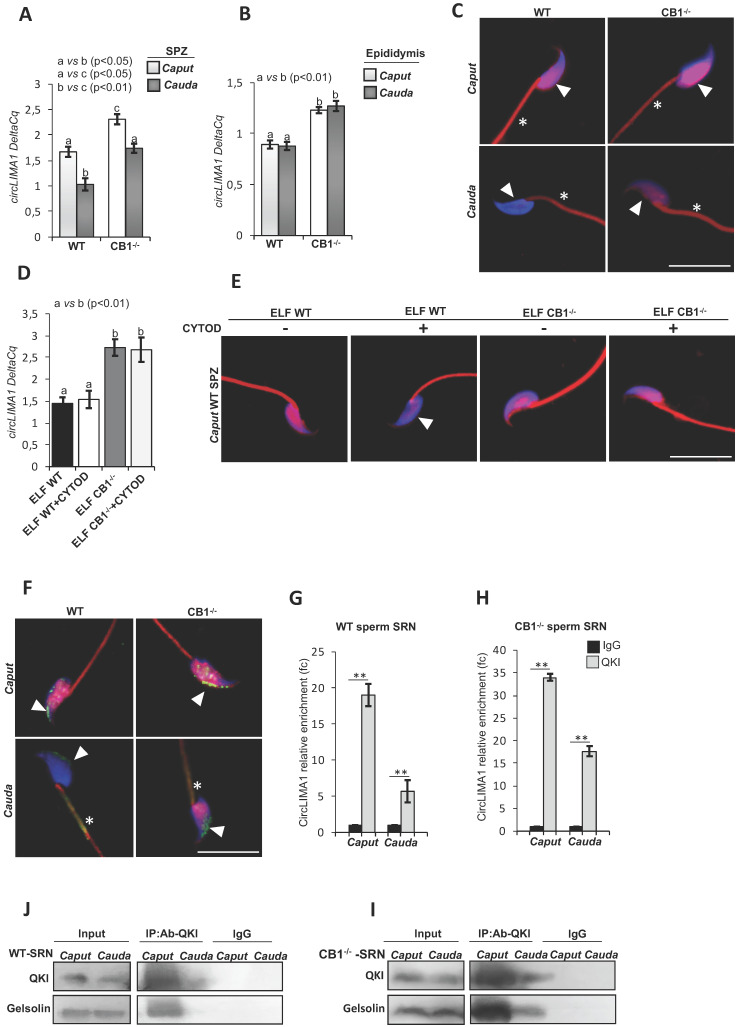
** CircLIMA1-dependent sperm nuclear actin organization along the epididymis.** qRT-PCR detection of circLIMA1 expression levels in *caput* and *cauda* SPZ (**A**) or epididymis (**B**) from WT and CB1^-/-^ mice. (**C**) Immunofluorescence analysis of F-actin by phalloidin staining in *caput* and *cauda* SPZ from WT and CB1^-/-^ mice. qRT-PCR detection of circLIMA1 expression levels (**D**) and Immunofluorescence analysis of F-actin by phalloidin staining (**E**) in *caput* SPZ from WT mice *in vitro* co-incubated with: WT *Caput* ELF (CTRL group), WT *Caput* ELF combined with Cytochalasin-D (C8273; Sigma-Aldrich, Milano, Italy) at the concentration of 10 μM (ELF WT+CYTOD); CB1^-/-^ *Caput* ELF (ELF CB1^-/-^); CB1^-/-^ *Caput* ELF combined with Cytochalasin-D 10 μM (ELF CB1^-/-^ +CYTOD); (n=3 different samples for each experimental group from 8 different animals in triplicate). qRT-PCR data are normalized using *Cyclophilin* and *RPS18*, for SPZ and epididymis respectively, expressed as DeltaCq and reported as mean value ± S.E.M. Experimental groups with statistically significant differences (p<0.05; p<0.01) were indicated with different letters; the experimental groups without statistically significant differences were indicated with the same letter. White full arrowheads and asterisks represent F-actin localization (red) in sperm head and tail, respectively. Nuclei were labeled with DAPI (blue). Scale bar: 15 µm (**F**) Immunofluorescence analysis of QKI protein in *caput* and *cauda* SPZ from WT and CB1^-/-^ mice. White full arrowheads and asterisks represent QKI localization (green) in sperm head and tail, respectively. Nuclei were labeled with DAPI (blue); F-actin was labeled with phalloidin staining (red). Scale bar: 15 µm (**G-H**) The enrichment levels of circLIMA1 in the products of RIP assay (QKI-IP compared with IgG-IP) in *caput* and *cauda* SRN from WT (**G**) and CB1^-/-^ (**H**) mice detected by qRT-PCR. Data are reported as mean ± SEM from three independent experiments. **p<0.01. (**J-K**) Western blot analysis of RIP protein fraction immunoprecipitated with QKI Ab (QKI-IP) in *caput* and *cauda* SRN from WT and CB1^-/-^ mice. QKI-IP was analyzed in comparison to control IgG-IP and Input protein extracts.

We extended our previous results and studied the hypothetical relationship between circLIMA1 and F-actin remodeling in SPZ transiting along the epididymis. Thus, the sperm samples from *caput* and *cauda* epididymis of WT and CB1^-/-^ mice were isolated and processed to analyze: *i*) circLIMA1 expression levels (Figure 5**A**) by qRT-PCR analysis and *ii*) F-actin signal (Figure 5**C**) by immunofluorescence analysis through phalloidin staining. In addition, to evaluate the epididymal contribution, we also analyzed circLIMA1 expression levels in *caput* and *cauda* epididymis of WT and CB1^-/-^ (Figure 5**B**).

Results showed that circLIMA1 levels significantly decreased (p<0.05) in SPZ from *caput* to *cauda* epididymis, in both WT and CB1^-/-^. Interestingly, both in *caput* and *cauda* epididymis, SPZ showed circLIMA1 levels significantly higher (p<0.05) in CB1^-/-^ mice when compared to WT (Figure 5**A**).

No difference was evident between *caput* and *cauda* epididymis in both WT and CB1^-/-^, although circLIMA1 levels were significantly higher in both *caput* and *cauda* epididymal tissue from CB1^-/-^ than in WT (Figure 5**B**).

The staining of phalloidin for F-actin was compared between *caput* and *cauda* SPZ, in both WT and CB1^-/-^ mice. In detail, phalloidin-DAPI co-localization showed that the F-actin was distributed in the nucleus as well as in sperm tail, with a decrease of phalloidin signal in SPZ from *caput* to *cauda* in both genotypes analyzed, limited to the nucleus (Figure 5**C**). In particular, CB1^-/-^ *cauda* SPZ showed a higher F-actin retention in comparison to WT *cauda* sperm, reflecting circLIMA1 expression and suggesting, in absence of CB1, a close association between circLIMA1 levels and a defective actin depolymerization in SPZ from *caput* to* cauda*.

To demonstrate the possible circLIMA1 involvement in F-actin organization pathway in SPZ from *caput* to* cauda*, we set up an *in vitro* experiment in order to assess, firstly, the sperm uptake of circLIMA1 from the Epididymal Luminal Fluid (ELF) *via* epididymosomes, and consequently, to correlate, in sperm, the cargo of circLIMA1 with the actin polymerization. Therefore, *caput* SPZ from WT mice were incubated with *caput* ELF of both WT and CB1^-/-^ (ELF WT and ELF CB1^-/-^ group, respectively) and processed to analyze: *i*) circLIMA1 expression levels (Figure 5**D**) by qRT-PCR analysis and *ii*) F-actin signal (Figure 5**E**) by immunofluorescence analysis through phalloidin staining.

We chose to use *caput* ELF from CB1^-/-^ as the previous results shown in Figure 5**B** indicated higher circLIMA1 levels in the CB1^-/-^ *caput* epididymis, without any difference with those of *cauda*.

The results showed that circLIMA1 levels increased in *caput* SPZ co-incubated with *caput* ELF from CB1^-/-^ (ELF CB1^-/-^ group) in comparison to ELF WT group (p<0.01; Figure 5**D**), suggesting that circLIMA1 was shuttled from *caput* ELF to SPZ. In addition, we treated WT *caput* sperm with WT and CB1^-/-^ *caput* ELF in combination with 10 μM Cytochalasin-D, a strong inhibitor of actin polymerization (ELF WT + CYTOD and ELF CB1^-/-^ + CYTOD group, respectively; Figure 5**D**).

Interestingly, CYTOD treatment did not change circLIMA1 levels in both experimental groups in comparison to related control groups (ELF WT and ELF CB1^-/-^ group, respectively).

As expected, ELF WT group showed an F-actin nuclear localization which disappeared following treatment with CYTOD (ELF WT + CYTOD group; Figure 5**E**).

Additionally, in condition of increased uptake of circLIMA1 (ELF CB1^-/-^ group) the F-actin nuclear signal was high and remained constant even after treatment with CYTOD (ELF CB1^-/-^ + CYTOD group; Figure 5**E**), suggesting a close relation between circLIMA1 and actin polymerization.

In order to clarify the molecular mechanism through which circLIMA1 may regulate nuclear actin depolymerization during epididymal transit, we focused our attention on QKI, the RBP reported to shuttle between the nucleus and the cytoplasm and closely involved in the regulation of several actin-filament cofactors. Interestingly, QKI immunefluorescence analysis, carried out in *caput* and *cauda* of both WT and CB1^-/-^ SPZ, showed an intriguing nuclear localization changing during epididymal transit (Figure 5**F**). In detail, results showed that QKI signal was mainly distributed in the nucleus with a clear decrease in *cauda* SPZ of both genotypes analyzed (Figure 5**F**). In addition, the reduction of QKI nuclear localization observed during epididymal transit was associated with a flagellar signal acquisition in *cauda* SPZ, suggesting a possible QKI shuttling from sperm nucleus to tail. Furthermore, CB1^-/-^ *cauda* SPZ showed a higher nuclear QKI retention in comparison with WT *cauda* sperm, suggesting a defective QKI shuttling involved in incomplete nuclear actin depolymerization observed in CB1^-/-^ *cauda* SPZ. Based on these observations, we isolated the Sonication-Resistant-sperm Nuclei (SRN) fraction from *caput* and *cauda* SPZ of both WT and CB1^-/-^, to investigate the hypothetical relationship between circLIMA1 and QKI at nuclear level during epididymal transit. To evaluate the physical interaction between QKI and circLIMA1, we carried out an RNA immunoprecipitation (RIP) assay in SRN from *caput* and *cauda* epididymis of both WT and CB1^-/-^, using QKI antibody (Figure 5**G-H**). The results showed a 19.0- and 5.7-fold enrichment of circLIMA1, relatively to the use of IgG control, in *caput* and *cauda* WT SRN, respectively (Figure 5**G**), whereas in CB1^-/-^ SRN a 34.0- and 17.7-fold enrichment of circLIMA1 was observed in* caput* and *cauda,* respectively (Figure 5**H**), reinforcing the idea that the direct interaction with QKI regulates circLIMA1 nuclear localization and in turn, F-actin depolymerization. In order to investigate a possible regulation exerted by QKI-circLIMA1 interactome on actin remodeling factors, we analyzed Gelsolin protein levels in RIP protein fractions, as particularly active in sperm actin remodeling. The western blot analysis on QKI immunoprecipitated proteins relative to RIP fractions evidenced a clear QKI-Gelsolin interaction in *caput* SRN of both WT and CB1^-/-^ that disappeared and persisted in *cauda* WT and CB1^-/-^ SRN, respectively (Figure 5**I-J**). The reduction of QKI-Gelsolin interaction was dependent on the variations of nuclear QKI content, but not of Gelsolin, which resulted constant from *caput* to *cauda,* as confirmed by the analysis on input samples (total lysates isolated before the immunoprecipitations) (Figure 5**I-J**).

## Discussion

In the process of fertilization, SPZ ensure a normal embryo development providing a finely established epigenetic signature useful for the zygote formation 8, 29. To perform this primary function, SPZ not only undergo, a dynamic histone displacement dependent chromatin maturation, but enrich themselves with a large cargo of RNAs constituted by both coding and non-coding RNAs 8, 30. In this scenario, circRNAs are acquiring an increasingly prominent role in the sperm quality setting, indeed a circRNA cargo has been previously characterized in human good and poor quality SPZ as well as in asthenozoospermic patients 12, 13. The activity of the endocannabinoid receptor CB1 is required for the production of SPZ with mature chromatin and epigenetic landscape as confirmed by CB1^-/-^ male mouse, an inefficient spermatogenesis model characterized by SPZ with uncondensed chromatin and elongated nuclear size comparable with poor quality human SPZ 6, 18, 20, 21. Interestingly, CB1 activity leads to an increase in sperm backsplicing capacity, suggesting a tight association between endocannabinoids and spermatic circRNA cargo establishing 6.

Using a microarray-based strategy, we profiled circRNA expression pattern in WT and CB1^-/-^ epididymal SPZ. CircRNA microarray analysis identified a total of 45 DE-circRNAs, consisting of 33 up-regulated and 12 down-regulated circRNAs in CB1^-/-^ compared to WT SPZ, respectively. Genome analysis showed that several chromosomes (chr3, chr5, chr20, chr21, chr22, chrY, chrM) did not show any DE-circRNAs. Interestingly, DE-circRNAs, up-regulated in CB1^-/-^ SPZ, showed a peculiar behavior as: *i*) DE-circRNAs, derived from host genes of the same gene family, have tethering activity toward the same miRNA, *ii*) more than two DE-circRNAs have tethering activity toward the same miRNA or different miRNA isoforms, suggesting a sophisticated regulation mechanism of down-stream mRNA targets exerted by spermatic circRNAs. As highlighted by GO functional enrichment analysis, DE-circRNAs in CB1^-/-^ SPZ appear involved in biological pathways underling actin filaments assembly, organization as well as remodeling.

Actin represents the most abundant cytoskeletal protein in the sperm head which actively participates not only to the correct sperm morphology, but also to fundamental spermatic functions such as acrosomal reaction and capacitation 31, 32. In fact, during spermiogenesis, the actin filaments located in the sub-acrosomal space participate to: *i*) the correct positioning and anchoring of neo-forming acrosome and *ii*) the nuclear remodeling of the spermatids 33, 34. Interestingly, in CB1^-/-^ mouse model, a compromised spermiogenesis, consisting of defective histone displacement and immature SPZ production, occurs 35. These intriguing associations led us to hypothesize that a complex crosstalk between circRNAs and actin dynamics could be defective in CB1^-/-^ male mouse; therefore, in this work, we have pointed the attention on circLIMA1 as its linear counterpart encodes for an actin binding protein involved in the positive regulation of actin filament polymerization 24. The experimental validation of circLIMA1 in testis, epididymis and SPZ has shown a strong increase of expression in CB1^-/-^ for all the districts investigated, strengthening our idea that an anomalous regulation of actin dynamics, circRNA-dependent, starts from the testis and persists during epididymal sperm maturation. In agreement, the construction of a ceRNET for circLIMA1, carried out to shed light on predicted mRNA targets, allowed to the identification of five miRNAs, all involved in the regulation of targets strongly active in the cytoskeleton regulation. In fact, among predicted mRNA targets, it has been well demonstrated the ability of FOS family members to regulate cell motility by altering the organization of the cytoskeletal actin 36 and the capacity of the actin filament crosslinking the protein AFAP1 to affect actin filament integrity 37. The validation of circLIMA1 dependent-ceRNET - carried out, firstly, assessing the ability of circLIMA1 to directly bind to miR-214-3p and miR-7a-5p by luciferase reporter assay strategy, and, then, evaluating the actin-cytoskeleton architecture state following mmu-miR-214 or mmu-miR-7a-5p miRNA mimics transfection in GC-2 cells - has clearly confirmed its cytosolic activity in the regulation of the cytoskeletal actin.

Considering that during epididymal transit SPZ undergo several post-testicular maturational events regarding: *i*) nuclear compaction, *ii*) cytoskeletal structure and *iii*) RNA/protein payload, we have decided to extend our investigation with the aim to explore a putative role of circLIMA1 in the epididymal sperm cytoskeletal actin maturation 38-40. Despite a similar reduction trend of circLIMA1 in both WT and CB1^-/-^ SPZ, indicative of a progressive loss during epididymal transit, CB1^-/-^ SPZ retain a higher circLIMA1 content than WT in both *caput* and *cauda*. Since epididymal sperm maturation provides several molecular changes useful for sperm functions such as capacitation and acrosome reaction, once again our attention has been directed on a close relation between circLIMA1 and actin filaments dynamic organization, considering that a fine temporal regulation of actin polymerization regulates hyperactivated motility acquisition and prevents spontaneous acrosomal reaction 31, 41. Interestingly, the decrease of F-actin observed from* caput* to *cauda,* in both WT and CB1^-/-^ SPZ, was only confined to the nuclear area and appeared defective in *cauda* CB1^-/-^ SPZ, reflecting circLIMA1 trend one. These data led us to think at circLIMA1 as a positive regulator of spermatic actin polymerization, able to specifically act at nuclear level.

With this in mind, we set an *in vitro* Cytochalasin-D experiment on WT SPZ, demonstrating that the co-incubation with CB1^-/-^ ELF, enriched of circLIMA1, effectively counteracted nuclear actin depolymerization through the sperm uptake of circLIMA1* via* epididymosomes.

At this point we asked two questions: *i*) how does circLIMA1, being an exonic circRNA, and therefore predominantly localized at cytoplasmic level, translocate to the nucleus? *ii*) circLIMA1 is able to regulate the polymerization of nuclear actin with a molecular mechanism different from the validated cytosolic ceRNET? To answer these questions, we have explored a potential ceRNET-independent pathway, focusing our attention on a possible physical interaction between circLIMA1 and QKI, an RBP able to regulate the expression of actin remodeling cofactors and, more interestingly, able to exert nucleo-cytosol shuttling 42, 43. Effectively, QKI sperm localization changed during epididymal transit, showing a clear shuttle from nucleus to flagellum, supporting the idea of its possible involvement in nuclear actin depolymerization observed from *caput* to *cauda* epididymis. In order to investigate circLIMA1/QKI interaction at nuclear level, avoiding the cytosolic QKI component, we carried out RIP experiments only in SRN sperm nuclei. We showed a strong physical interaction between circLIMA1 and QKI, which gradually decreases in *cauda* SRN, concomitantly with the shuttle of QKI in the flagellum. Surprisingly, once again, this trend was also confirmed in CB1^-/-^, but appears less efficient given the abnormal presence of QKI and circLIMA1 in *cauda* CB1^-/-^ SRN, which well matched with the not proper depolymerization of nuclear actin. These data suggested a new putative role of circLIMA1 as molecular adaptor useful for QKI activity. This hypothesis is consistent with the recent findings that have clarified new aspects of RNA molecules, different from the canonical features as RNAs can adopt diverse architectures: *i*) to recognize and modulate protein function, *ii*) to provide landing sites for gene control factors, *iii*) to mimic an open DNA promoter complex to inhibit RNA transcription, *iv*) to recognize members of drug classes and fundamental metabolites 44-48.

Lastly, we investigated the Gelsolin as an actin regulator candidate to be tethered by QKI-circLIMA1 interactome. Gelsolin acts as a negative factor on the actin polymerization and its inactive form favors F-actin formation during sperm capacitation and motility increase 31, 49. Interestingly, Gelsolin-QKI interaction occurred only in *caput* SRN, completely disappearing in *cauda* WT SRN, whereas it persisted in *cauda* CB1^-/-^ SRN. It is likely that the loss of interaction with QKI, that in cooperation with circLIMA1 acts as a Gelsolin molecular inhibitor, makes physically available an active form of Gelsolin needful for actin depolymerization starting through; this molecular mechanism appears inefficient in *cauda* CB1^-/-^ SPZ due to the persistent Gelsolin-QKI interaction and the failure of QKI flagellum translocation. According to this hypothesis, recent findings regarding the functional role of actin polymerization on sperm motility have shown that the acquisition of hyperactivated motility during capacitation requires Gelsolin activation only in the sperm nucleus, in order to prevent actin depolymerization in the flagellum 49. Considering that SPZ acquire the progressive motility during epididymal maturation, a similar Gelsolin regulation along the epididymis is not to be excluded 50. Overall, the results obtained demonstrate that the endocannabinoid system is potentially involved in circRNA biogenesis as *Cb1* gene deletion interferes with the normal sperm circRNAs cargo, inducing a differential expression profile in CB1^-/-^ SPZ. In addition, we have identified in circLIMA1 a potential regulator of actin polymerization highlighting: *i*) a remodeling mechanism of sperm nuclear actin occurring along the epididymal transit, *ii*) a novel circRNA biological pathway, ceRNET-independent.

Considering that F-actin modulation is required for fully functional dynamism of male and female pronuclei 51, circLIMA1 could be considered a new fascinating key actor involved in the regulation of nuclear spermatic F-actin needful for a successful embryo development. Furthermore, the increased levels of circLIMA1 in CB1^-/-^ SPZ, associated with an inefficient depolymerization of nuclear actin, specifically illustrate how endocannabinoids, by regulating circRNA cargo, may contribute to sperm morpho-cellular maturation.

## Supplementary Material

Supplementary figures.

## Figures and Tables

**Table 1:** Primer Sequences and Annealing Temperatures

Gene Primers	Sequences 5′-3′	Tm (°C)
*Mmu circ-Mettl9* S	GCATTGGTTTTGCCCTTTCA	53
*Mmu circ-Mettl9* AS	ACAGACTGAAGTGATTCGCA	
*Mmu circ-LIMA1* S	TTCTCCTTCCAAAAACCCTGA	54
*Mmu circ-LIMA1* AS	CTGTTTGGCAGTGAGTCTGT	
*Mmu circ-KMT2C* S	GCTCCTGAAAGATGCCTCG	56
*Mmu circ-KMT2C* AS	TTTCTGTTTCCGTCGTCTCC	
*Mmu circ-PSMA8* S	ATACCCAGAGCAATGGACGA	53
*Mmu circ-PSMA8* AS	CATCCGCTGTCAGACTTTCC	
*Mmu circ-RBM39* S	ACTTAAACAATACTTGCCTAACGA	52
*Mmu circ-RBM39* AS	GACCACGGTACAGCGATAAG	
*Mmu circ-ASUN* S	ATTCCTATGAAGTGATAGTCATGT	52
*Mmu circ-ASUN* AS	GCAGCAAGCTTGTTATGCTC	
*Mmu circ-TLK2* S	AGGCATTTGATCTAACGGAGCA	53
*Mmu circ-TLK2* AS	AATCACAGTTGGCTTGTGGT	
*Mmu circ-DLC1* S	GAGTCCACCGATCCATCATC	56
*Mmu circ-DLC1* AS	TCCCTTGTTTCCATGGCTTT	
*Mmu circ-GNB1L* S	TCCCTGCAGGACTGCATTTAC	53
*Mmu circ-GNB1L* AS	GGGCAGAAATGGAGCGTGTT	
*Mmu circ-SPATA16* S	GCACACAACTGAATATACCCAAG	52
*Mmu circ-SPATA16* AS	ATTGCTGGGGAGGAAAACAC	
*Mmu circ-CCDC40* S	ATGGATGCGCTCAGTGG	53
*Mmu circ-CCDC40* AS	CACCTGCTTCATGACTTGGA	
*Mmu circ-HDAC8* S	TTGCTGGACATACTTGACCG	54
*Mmu circ-HDAC8* AS	TGCTTCATCTCTCATGATCTGG	
*Mmu circ-SATB2* S	AACCGCACACAGGTTTGAT	52
*Mmu circ-SATB2* AS	AAAAGCACATCTTTCCGCAC	
*Mmu circ-ZCCHC2* S	TCAGAGTCACAAAGTCCGCAG	52
*Mmu circ-ZCCHC2* AS	TCCTGTAGTTCTTGGTGGGTTTT	
*Mmu circ-PARP14* S	TCTCAAATCAGGGGTCTCCA	53
*Mmu circ-PARP14* AS	GCATGGGTCCAAGAGAGAAC	
*Mmu circ-DAGLB* S	TCCACCAGCAACAAGACAAT	54
*Mmu circ-DAGLB* AS	TGACAATCCACCTGAGGCT	
*RPS18* S	GAGACTCTGGATGCTAACTAG	56
*RPS18* AS	GGACATCTAAGGGCATCACAG	
*Cyclophilin-A* S	TGGTCTTTGGGAAGGTGAAAG	52
*Cyclophilin-A* AS	TGTCCACAGTCGGAAATGGT	
