# Supplementary material for: Actin remodeling driven by circLIMA1: sperm cell as an intriguing cellular model
Source: Int J Biol Sci. 2022 Aug 8;18(13):5136–53. doi: 10.7150/ijbs.76261 (PMC9379403; doi:10.7150/ijbs.76261)
Supplement: Supplementary file 1 — Supplementary figures. [file ijbsv18p5136s1.pdf]

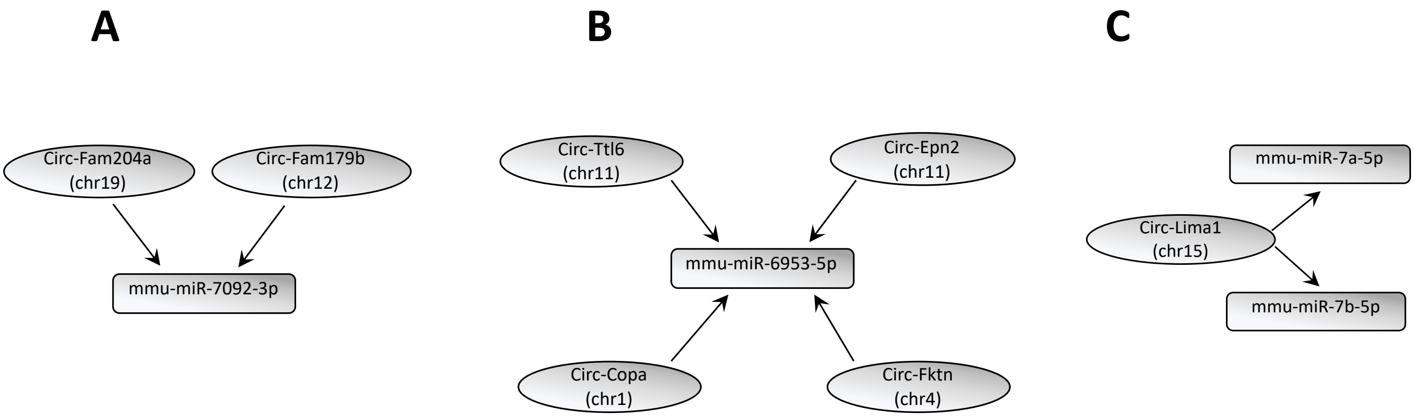

Supplementary Figure S1

Figure S1. Functional clustering of DE-circRNAs in CB1$^{-/-}$ compared to WT SPZ. **(A)** Two circRNAs whose host genes are members of the same gene family but located on different chromosomes show tethering activity toward the same miRNA as target. **(B)** Four circRNAs whose host genes are located on different chromosomes, tether the same miRNA. **(C)** The same circRNA shows tethering activity toward a family of miRNA isoforms. Networks were built using Cytoscape. Ovoid and rectangular symbols represent circRNAs and miRNAs, respectively. The arrow indicates the tethering activity of circRNAs toward miRNAs.

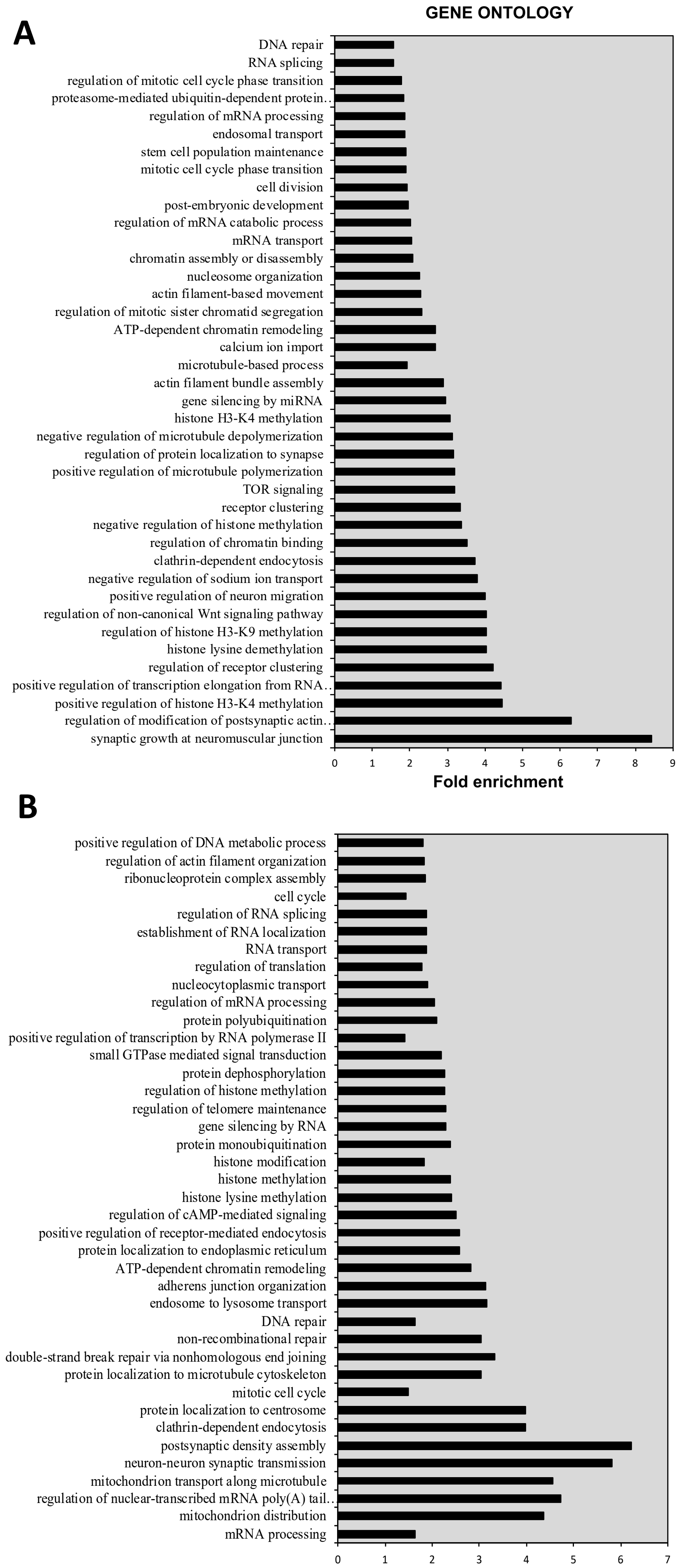

Supplementary Figure S2

Figure S2. Gene ontology (GO) enrichment analysis of host genes. **(A)** The Top 40 GO annotations in $CB1^{-/-}$ SPZ up-regulated circRNAs. **(B)** The Top 40 GO annotations in $CB1^{-/-}$ SPZ down-regulated circRNAs.

A

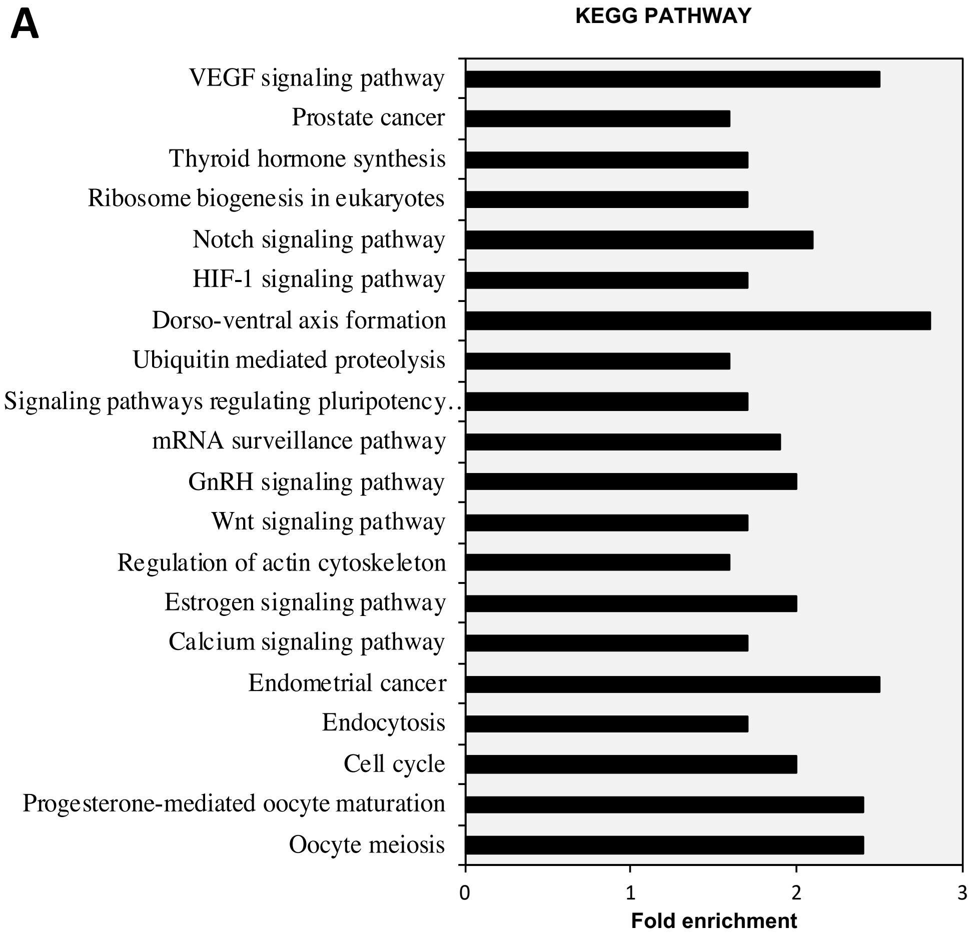

B

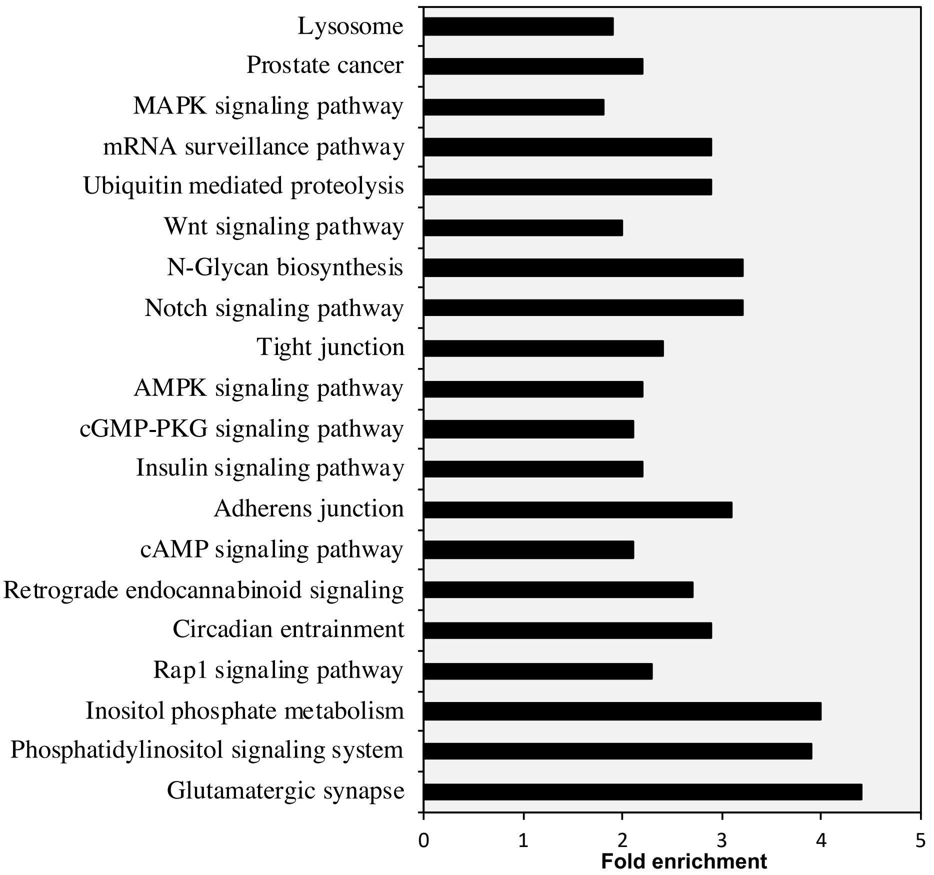

Supplementary Figure S3

Figure S3. Kyoto Encyclopedia of Genes and Genomes (KEGG) pathway annotation of host genes. **(A)** The Top 20 KEGG signaling pathway annotations in $CB1^{-/-}$ SPZ up-regulated circRNAs. **(B)** The Top 20 KEGG signaling pathway annotations in $CB1^{-/-}$ SPZ down-regulated circRNAs.
